# Supplementary material for: Origin of charge transfer and enhanced electron–phonon coupling in single unit-cell FeSe films on SrTiO3
Source: Nat Commun. 2017 Aug 9;8:214. doi: 10.1038/s41467-017-00281-5 (PMC5548863; doi:10.1038/s41467-017-00281-5)
Supplement: Supplementary file 1 — Supplementary Information [file 41467_2017_281_MOESM1_ESM.pdf]

File name: Supplementary Information
Description: Supplementary Figures, Supplementary Notes and Supplementary References

File name: Peer Review File
Description:

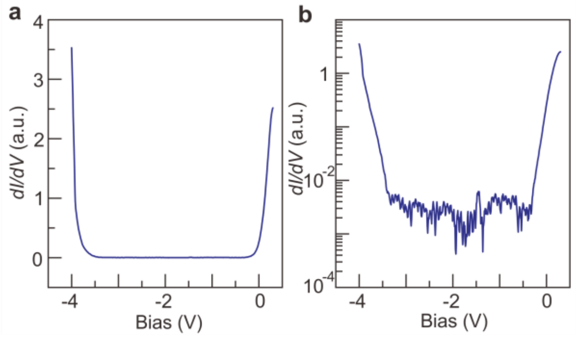

Supplementary Figure 1. Low-temperature tunneling spectrum of pristine STO. (**a**) Typical dI/dV of 0.5 wt % Nb-doped STO. (**b**) Logarithm of (**a**). The Nb-doped STO measured here was annealed at 1200 °C following the same treatment as the substrates used for the growth of FeSe.

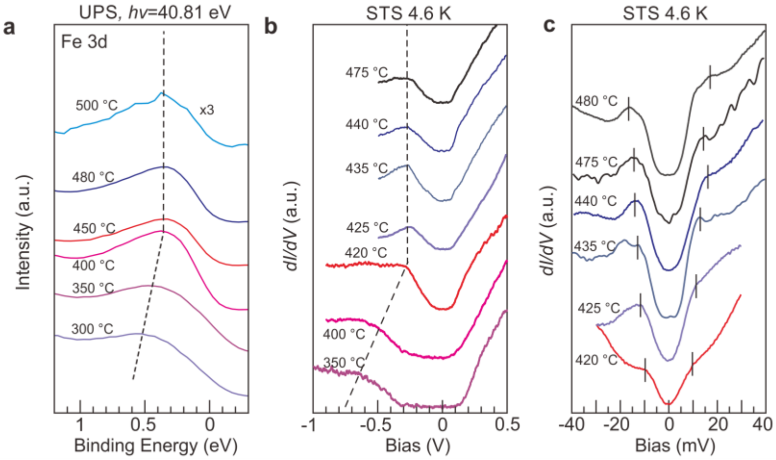

Supplementary Figure 2. Correlation between annealing and superconductivity. (**a**) Fe 3*d* peak of 1uc-FeSe/STO at different annealing stages revealed by He-II light with a photon energy of 40.81 eV. (**b**-**c**) Low-temperature STS data obtained from a separate system. Here 1uc-FeSe was grown on 0.05 wt% Nb-doped $SrTiO_3$. At annealing temperatures higher than 420 °C, a superconducting gap starts to open. Vertical bars in **c** mark the coherence peak positions taken for calculating the superconducting gap shown in Fig. 1c.

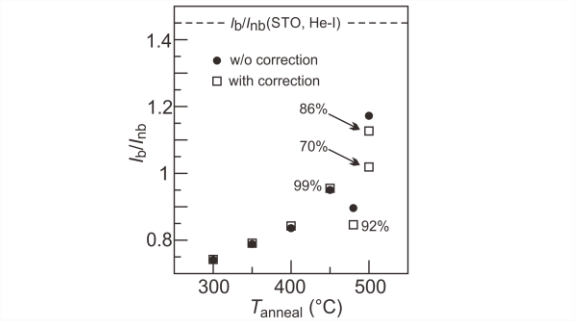

Supplementary Figure 3. Intensity ratio before and after corrections. We take into account the intensity contribution from 1uc-FeSe and the exposed substrate if it exists. For the data point at 500 °C, even by assuming a low coverage of 70%, the bonding to non-bonding peak ratio after correction still exceeds the values at lower annealing temperature.

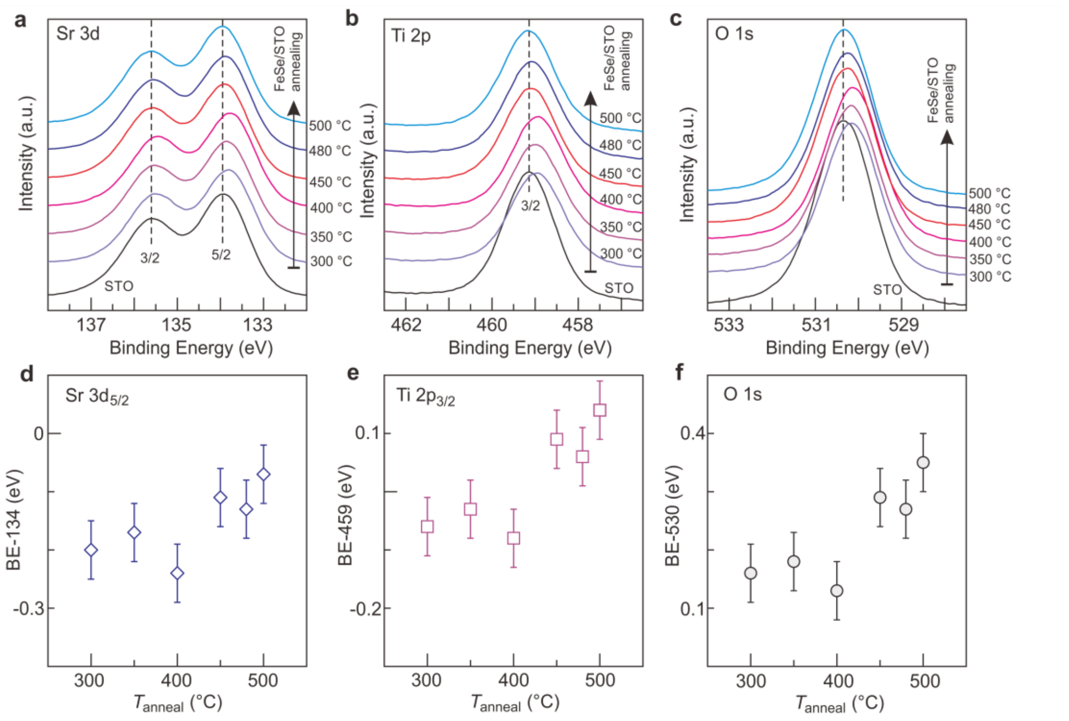

Supplementary Figure 4. XPS spectra of Sr, Ti, O and the peak positions. 1uc-FeSe/STO films were annealed at 300, 350, 400, 450, 480 and 500 °C for two hours each. Peak positions were determined by fitting the Gaussian-Lorentzian function with a Shirley nonlinear sigmoid-type baseline subtracted (see Supplementary Figure 9). Error bars were estimated by considering the energy resolution of the measurement.

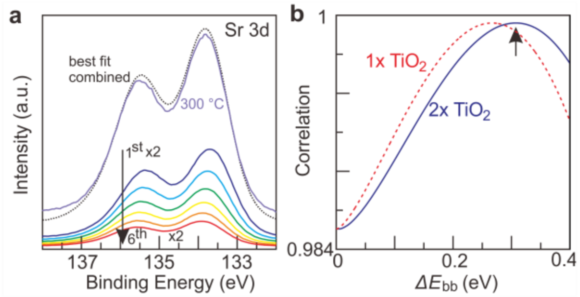

Supplementary Figure 5. Simulation and correlation calculation for Sr 3*d* spectrum at T_{anneal} = 300 °C. (**a**) Simulated spectra of the first 6th layers of SrO with the fitted band bending value (see the texts for the fitting procedure). Dotted curve is the total contribution from 100 layers of TiO_2/SrO, where only the SrO layers contribute to Sr 3*d* spectrum here. Solid purple curve is the experimental data. (**b**) Calculated correlation between the experimental curve at 300 °C and the fitted spectrum as a function of the band bending value. Black arrow marks the best correlation. The simulated spectra in Fig. 2c and Supplementary Fig. 7a are obtained by considering the recently established layer-by-layer structure of STO close to the FeSe/STO interface. Especially, double TiO_2 layers at the interface have been experimentally identified[2,7]. Panel **b** here compares the calculated correlations considering a single TiO_2 layer and double TiO_2 layer structures.

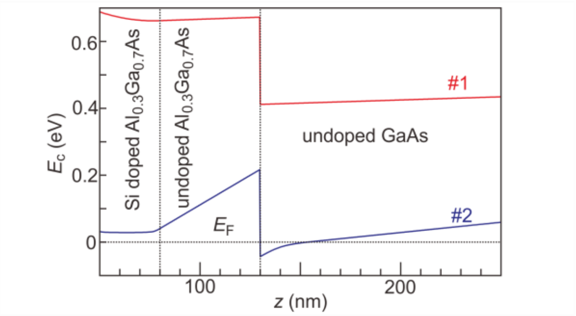

Supplementary Figure 6. Conduction band profile of the AlGaAs/GaAs heterostructure. The heterostructure follows the reported one[8] but with two doping concentrations. The band diagram is calculated by using a self-consistent Poisson-Schrodinger solver (Greg Snider, University of Notre Dame. https://www3.nd.edu/~gsnider/). Increasing the doping concentration dramatically pushes down the conduction band and results in a triangular shaped barrier in the undoped AlGaAs region.

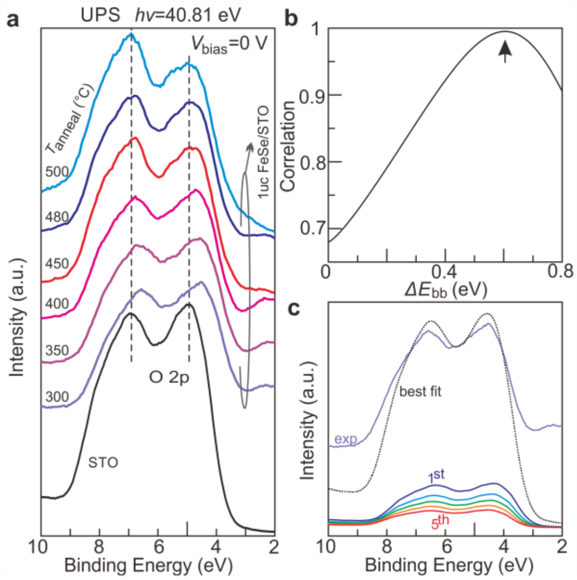

Supplementary Figure 7. O 2*p* valence band spectra. (**a**) UPS spectra of O 2*p* features obtained by using the He-II light. (**b**) Calculated correlation between the experimental curve at 300 °C and the fitted spectrum as a function of the band bending value. (**c**) Comparison between the experimental curve and the best fit curve. The simulated curve employs the optimized band bending value showing the maximum correlation. Intensity contributions from the first five layers are also shown.

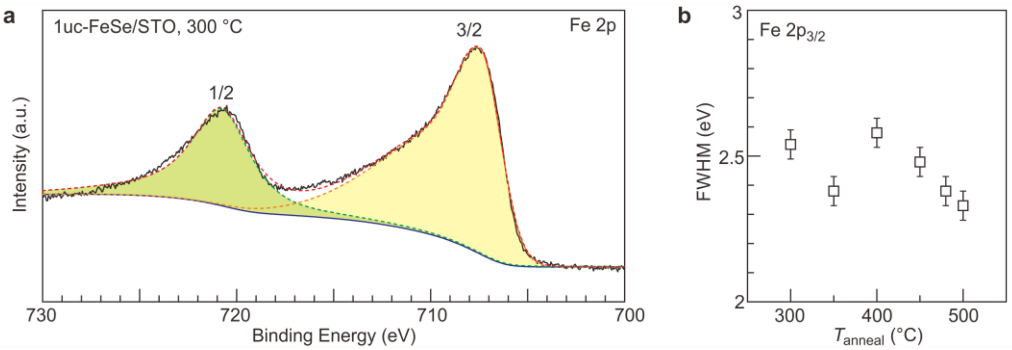

Supplementary Figure 8. Fitting of a Fe 2*p* spectrum and the fitted full width at half maximum (FWHM) of the Fe 2$p_{3/2}$ peak. (**a**) XPS spectrum of Fe 2*p* obtained from 1uc-FeSe/STO at 300 °C. This spectrum is fitted by Gaussian-Lorentzian function with the Shirley background (gray curve) subtracted. (**b**) FWHM of the Fe 2$p_{3/2}$ peak at different annealing stages. The peak width slightly narrows with annealing, reflecting the renormalization effects.

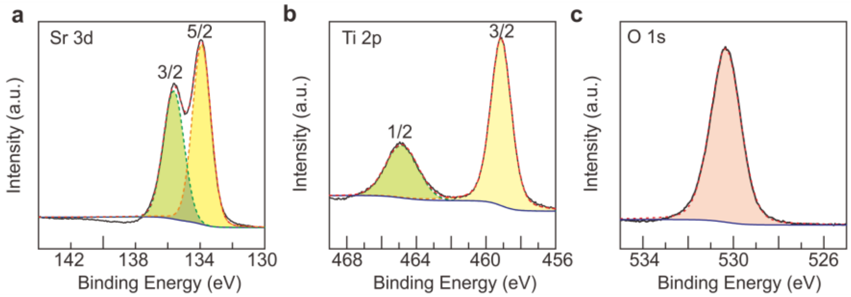

Supplementary Figure 9. XPS spectra for pristine $SrTiO_3$ before FeSe deposition. (**a-c**) XPS spectra for Sr 3*d*, Ti 2*p* and O 1*s* and corresponding fitting by Gaussian-Lorentzian function after subtracting a Shirley background. Solid and dashed lines are experimental data and fitted curves, respectively. Blue lines represent the Shirley nonlinear sigmoid-type baseline.

Supplementary Note 1. Correction of the intensity ratio of the bonding over non-bonding peak.

In the main text, we estimate the intensity ratio I_b/I_{nb} simply from the normalized intensities of the two peaks. However, there are two contributions other than that from the covered STO. First, the spectrum of the 20uc-FeSe/STO has higher intensity in the energy range of 4 to 6 eV (where the non-bonding peak of STO situates) than that in the energy range of 6 to 8 eV. Although a single layer of FeSe has a reduced contribution, this effect may still devaluate the I_b/I_{nb} ratio from the covered STO. Secondly, at T_{anneal} = 450 °C, 480 °C and 500 °C, the FeSe coverage becomes 99%, 92% and 86%. The exposed STO may therefore contribute an increase of the bonding peak height, since the bonding peak in pristine STO has a higher intensity than that of the nonbonding peak, as disclosed in Fig. 1b. In order to extract I_b/I_{nb} of the covered STO only, we subtract the two above mentioned contributions by assuming: (1) the spectrum purely from 1uc-FeSe is similar to that of 20uc-FeSe but with a lower intensity. (2) the exposed STO shows the same spectrum as that of STO before depositing FeSe.

The spectrum of 1uc-FeSe/STO sums up both the FeSe covered region and the exposed region:

$$I_{\mathrm{1FeSe/STO}} = xI_{\mathrm{1FeSe}} + xI_{\mathrm{STO},c} + (1-x)I_{\mathrm{STO}}, \tag{1}$$

where x represents the FeSe coverage, I_{1FeSe} is the intensity contribution solely from the single FeSe layer. $I_{\mathrm{STO},c}$ and I_{STO} denote the contributions from the covered and exposed STO, respectively.

Since in experiment, we employ the normalized spectrum by dividing the raw spectrum by the integrated intensity in the energy range dominated by the O 2p level (3.5 eV to 9.5 eV), the experimentally measured spectrum is (we use the upper index N to denote the normalized ones):

$$I^N_{\mathrm{1FeSe/STO}} = \frac{I_{\mathrm{1FeSe/STO}}}{\int_{\mathrm{O}\ 2p} I_{\mathrm{1FeSe/STO}} dE} = \frac{xI_{\mathrm{1FeSe}} + xI_{\mathrm{STO},c} + (1-x)I_{\mathrm{STO}}}{x\int_{\mathrm{O}\ 2p} I_{\mathrm{1FeSe}} dE + x\int_{\mathrm{O}\ 2p} I_{\mathrm{STO},c} dE + (1-x)\int_{O\ 2p} I_{\mathrm{STO}} dE}. \tag{2}$$

After some manipulations, we obtain:

$$I^N_{\mathrm{1FeSe/STO}} = \frac{x\beta I^N_{\mathrm{1FeSe}} + x\gamma I^N_{\mathrm{STO},c} + (1-x)I^N_{\mathrm{STO}}}{x\beta + x\gamma + (1-x)}, \tag{3}$$

where we define: $\beta = \frac{\int_{\mathrm{O}\ 2p} I_{\mathrm{1FeSe}} dE}{\int_{\mathrm{O}\ 2p} I_{\mathrm{STO}} dE}$ and $\gamma = \frac{\int_{\mathrm{O}\ 2p} I_{\mathrm{STO},c} dE}{\int_{\mathrm{O}\ 2p} I_{\mathrm{STO}} dE}$.

By comparing the intensities of the Fe 3*d* peak from 20uc-FeSe/STO and 1uc-FeSe/STO (Fig. 1b), we obtain that: $I^N_{\mathrm{1FeSe}} \approx 0.25 \times I^N_{\mathrm{20FeSe}}$. We estimate β by taking into account the cross sections of Fe 3*d* and O 2*p* at the ionization energy of He-I light: $\sigma^{\mathrm{Fe}3d} = 4.833$ and $\sigma^{\mathrm{O}2p} = 10.67$ MegaBarns(Mb)[1] and also the fact that the STO substrate is much thicker than FeSe:

$$\beta = \frac{\sigma^{\mathrm{Fe}3d}}{\sigma^{\mathrm{O}2p} \times \sum_{m=0}^{\infty} \exp\left(-\frac{m a_{\mathrm{STO}}}{2\lambda}\right)} = 0.08.$$

On the other hand, γ mainly reflects the damping of the intensity from the covered STO due to the presence of 1uc-FeSe: $\gamma = \exp\left(-\frac{d}{\lambda}\right) = 0.54$, where *d*=0.6 nm is the distance from the top of FeSe to STO[2]. We choose λ to be 1 nm based on the universal curve of the photoemission spectrum[3]. We can therefore obtain the spectrum from the FeSe covered STO:

$$I^N_{\mathrm{STO},c} = \frac{[x\beta + x\gamma + (1-x)] I^N_{\mathrm{1FeSe/STO}} - 0.25 x\beta I^N_{\mathrm{20FeSe}} - (1-x) I^N_{\mathrm{STO}}}{x\gamma}. \quad (4)$$

By knowing the bonding and nonbonding peak intensities: $I^N_{\mathrm{1FeSe/STO}}(b)$ and $I^N_{\mathrm{1FeSe/STO}}(nb)$ and the intensities at the corresponding energies from the spectra of the 20uc-FeSe/STO and pristine STO, one can calculate: $I^N_{\mathrm{STO},c}(b)/I^N_{\mathrm{STO},c}(nb)$ according to Supplementary Eq. (4). Supplementary Figure 3 shows the intensity ratio before and after such a correction. The corrected result still exhibits an increasing trend of the bonding to nonbonding ratio.

Supplementary Note 2. Quantitative determination of the band bending.

To start with, we assume no band bending in STO before the growth of FeSe. Such an assumption is valid since the Fermi level is measured to be close to the conduction band instead of pinned in the middle of the gap (see Supplementary Fig. 1). The spectrum of Sr 3*d*, Ti 2*p*, or O 1*s* after the growth of FeSe can be simulated by adding the intensity contribution layer-by-layer in the direction normal to the interface:

$$I^{\mathrm{Sr,Ti,O}}_{\mathrm{FeSe/STO}}(E) = \frac{\sum_m^N I^{\mathrm{Sr,Ti,O}}_{\mathrm{STO}}(E + \Delta E_m) \times e^{-\frac{z_m}{\lambda}}}{\sum_m^N e^{-\frac{z_m}{\lambda}}}. \quad (5)$$

Here $I^{\mathrm{Sr,Ti,O}}_{\mathrm{STO}}(E)$ is the spectrum of Sr 3*d*, Ti 2*p*, or O 1*s* from the pristine STO (Curves in the bottom of Supplementary Figs. 4a-c). The last term in the numerator reflects the exponential damping of the layers: z_m is the distance from the *m*-layer to the surface; $\lambda = \lambda^{\mathrm{Sr,Ti,O}}_{\mathrm{IMFP}} \cos\theta$ represents the effective inelastic mean free path (IMFP) at the emission angle of θ (θ = 37° for

XPS, and 29° for UPS). We use $\lambda_{\mathrm{IMFP}}^{\mathrm{Sr}\,3d} = 1.9$ nm, $\lambda_{\mathrm{IMFP}}^{\mathrm{Ti}\,2p} = 1.4$ nm, and $\lambda_{\mathrm{IMFP}}^{\mathrm{O}\,1s} = 1.3$ nm following the TPP-2M formula with the input material parameters of STO[4], for the UPS data we use $\lambda_{\mathrm{IMFP}}^{\mathrm{O}\,2p} = 1$ nm based on the universal curve of photoemission spectroscopy[3]. The band bending at each layer m is represented by the quantity ΔE_m. We use the simplified form: $\Delta E_m = \Delta E_{bb}\left(\frac{z_m}{L_{bb}} - 1\right)^2$ such that the top-most layer has the largest band bending ΔE_{bb} and it decays quadratically into the bulk. Instead of making L_{bb} a fitting parameter[5], we estimate the length of the bent region by the formula used in the metal/oxide/semiconductor heterostructures[6]: $L_{bb} = \sqrt{\frac{2\varepsilon_r\varepsilon_0\Delta E_{bb}}{(e^2 N_D)}}$. Here N_D is the doping concentration and is estimated to be 10^{21} cm^{-3}, ε_r is the relative dielectric constant of STO and is 300 at room temperature. For the STO with double TiO_2 layer at the interface, we use the following structural sequence based on the reported values[2]: TiO_2, $z_1 = 0$ nm; TiO_2, $z_2 = 0.24$ nm; SrO, $z_3 = 0.42$ nm; TiO_2, $z_4 = 0.6$ nm; SrO, $z_5 = z_4 + 0.195$ nm; …. For the case of a single TiO_2 layer at the interface, we use: TiO_2, $z_1 = 0$ nm; SrO, $z_2 = 0.195$ nm; TiO_2, $z_3 = 0.39$ nm; SrO, $z_4 = 0.39 + 0.195$ nm; ….

We then obtain ΔE_{bb}— the only fitting parameter—by finding the best correlation between the simulated and the experimental spectra. Supplementary Figure 7 shows an example by fitting the experimental curve at 300 °C. The calculated correlation as a function band bending values is shown for the two structural cases.

Supplementary Figure 7 summarizes the O 2*p* peaks observed by a He-II light. Supplementary Figure 7b shows the calculated value of correlation for each band bending when fitting the 300 °C curve. Supplementary Figure 7c displays the best fitting in comparison with the experimental data.

Supplementary References:

1. Yeh, J. J. & Lindau, I. Atomic subshell photoionization cross sections and asymmetry parameters: $1 \leqslant Z \leqslant 103$. At. Data. Nucl. Data Tables **32**, 1-155 (1985).

2. Li, F. *et al*. Atomically resolved FeSe/$SrTiO_3$(001) interface structure by scanning transmission electron microscopy. *2D Mater*. **3**, 024002 (2016).

3. Takayama, A. High-resolution spin-resolved photoemission spectroscopy and the Rashba effect in bismuth thin films. Chapter 2, 15-30, Springer, 2014.

4. Tanuma, S. *et al*. Experimental determination of electron inelastic mean free paths in 13 elemental solids in the 50 to 5000 eV energy range by elastic-peak electron spectroscopy. *Surf. Interface Anal*. **37**, 833 (2005).

5. Yoshimatsu, K. *et al*. Origin of metallic states at the heterointerface between the band insulators $LaAlO_3$ and $SrTiO_3$. *Phys. Rev. Lett.* **101**, 026802 (2008).

6. Sze, S. M. Physics of semiconductor devices, New York. Wiley, 1981.

7. Zou, K. *et al.* Role of double TiO_2 layers at the interface of FeSe/$SrTiO_3$ superconductors. *Phys. Rev. B* **93**, 180506 (2016).

8. Tsui, D. C., Stormer, H. L. & Gossard, A. C. Two-dimensional magnetotransport in the extreme quantum limit. *Phys. Rev. Lett.* **48**, 1559 (1982).
